# A Study of Traditional Chinese Medicine Body Constitution Associated with Overweight, Obesity, and Underweight

**DOI:** 10.1155/2017/7361896

**Published:** 2017-10-19

**Authors:** Mihui Li, Shuming Mo, Yubao Lv, Zihui Tang, Jingcheng Dong

**Affiliations:** ^1^Department of Integrative Medicine, Huashan Hospital, Fudan University, Shanghai, China; ^2^The Institutes of Integrative Medicine, Fudan University, Shanghai, China; ^3^Department of Integrative Medicine, Huashan Hospital North, Fudan University, Shanghai, China

## Abstract

**Objective:**

The aim of the study was to investigate the associations among the nine types of body constitution in traditional Chinese medicine (TCM) with the outcomes of overweight, obesity, and underweight.

**Method:**

Participants aged 30 to 90 years were recruited from communities in Shanghai and assessed using a self-administered questionnaire pertaining to their demographics, lifestyles, and self-reported medical history. The data of 3748 participants with complete information was available for the analysis. Multinomial logistic regression (MLR) analysis was performed to determine the associations among the TCM constitution variables and the health outcomes.

**Results:**

The standards of classification and determination of the constitution in TCM were used to gauge the patients' constitution type. MLR revealed independent and significant associations among the Qi_Deficient and Yang_Deficient groups with the outcomes of overweight, obesity, and underweight (*P* < 0.10 for all). MLR revealed independent and significant associations among the Qi_Deficient and Yang_Deficient groups with the outcomes of overweight, obesity, and underweight (*P* < 0.05 for all).

**Conclusion:**

Our study revealed significant negative correlations between the Qi_Deficient and Yang_Deficient groups with the outcomes of overweight, obesity, and underweight. On the other hand, positive correlations were found between Phlegm_Dampness and the outcomes of overweight and obesity.

## 1. Introduction

Recently, the systematic constitutional theory of traditional Chinese medicine (TCM) was proposed, and improvements have been made to it gradually. In TCM, the body constitution is an individual character compatible with nature and the social environment; formed in the process of human growth and development, one's body constitution indicates one's susceptibility to diseases, metabolism, and response to stimuli. Under TCM, there are nine constitutions: Neutral, Qi_Deficient, Yang_Deficient, Yin_Deficient, Phlegm_Dampness, Blood_Stasis, Qi_Stagnation, and Special [1]. The standards pertaining to the classification and determination of the constitution in TCM were issued by the China Association for Traditional Chinese Medicine. Based on the symptoms presented, it is possible to evaluate one's constitution using the standard questionnaire [2].

The body mass index (BMI), which is derived from the mass and height of an individual, is an attempt to quantify the amount of tissue mass in an individual and accordingly categorize that person as being underweight, overweight, obese, or of normal weight. Obesity is a leading preventable cause of death worldwide [3] and is associated with various health issues, particularly cardiovascular diseases, type 2 diabetes mellitus (DM), certain types of cancer, and asthma [3, 4]. The implications of the overweight health outcome are more controversial. Overweight can reduce life expectancy and increase the risk of oligospermia and azoospermia in men [5, 6]. However, overweight was not associated with excess mortality [7]. Underweight is considered unhealthy and is an established risk factor for osteoporosis, even among young people [8].

Constitutional consideration not only determines the patient's susceptibility to certain pathogens and diseases but is also closely related to the development, treatment, and prognosis of diseases [9]. When an individual's constitution is evaluated, the congenital hereditary factor, physiological functions, psychological conditions, and social factor are usually taken into account, suggesting that the constitution has a close relationship with chronic diseases. Several studies have suggested that the constitution under TCM relates closely to chronic diseases, such as obesity, type 2 DM, hypertension (HTN), and metabolism syndrome (MetS) [10–13]. However, few studies have systemically explored the relationship among the nine constitutions and overweight, obesity, and underweight. Based on previous studies, we assume that the constitutions may be associated with BMI. This study was aimed at investigating the relationship of body constitutions of TCM with overweight, obesity, and underweight in this sample.

## 2. Methods

As was mentioned earlier [14], we conducted a work to investigate the associations among chronic diseases and the nine body constitutions under TCM in a Chinese sample. We recruited more than 4,000 participants (30–90 years) from communities in Shanghai between 2011 and 2014. Participants with severe renal and hepatic function abnormalities and pregnant women were excluded. We obtained written consent from all participants before this study, which was approved by Ethical Committee of the Huashan Hospital and performed according to ethical standards in the Declaration of Helsinki. After excluding the incomplete responses, 3748 subjects with available data were used for the analysis.

All participants were completely evaluated for baseline characteristics. Similar to our previous studies [14–16], the baseline data consisted of demographical information, lifestyle, and medical history. Hypertension (HTN), BMI, and DM were defined as in earlier studies [14–16]. According to Chinese criteria, BMI (weight/height^2^, kg/cm^2^) was classified: underweight (<18.0 kg/m^2^), normal weight (≥18.0 kg/m^2^ and <24.0 kg/m^2^), overweight (≥24.0 kg/m^2^ and <28.0 kg/m^2^), and obesity (≥28.0 kg/m^2^) [17]. The TCM constitutions were assessed using a standard questionnaire recommended by the China Association for Traditional Chinese Medicine (Supplementary Document 1 in Supplementary Material available online at https://doi.org/10.1155/2017/7361896) [1].

### 2.1. Statistical Analysis

Differences analyses among participants categorized by BMI were performed using a one-way analysis of variance or *χ*^2^ test. Firstly, the association of the TCM constitution variables with the health outcomes was determined by using univariate regression analysis. Further, multinomial logistic regression (MLR) was employed to include a shared reference of normal weight in determining the associations among the constitutions with overweight, obesity, and underweight, control for potential confounding factors. The results were reported by using professional statistical software (Statistical Package for Social Sciences 16.0, SPSS, Chicago, IL, USA). A *P* value of <0.05 was considered significant (two-sided tests). Odds ratios (OR) with 95% confidence intervals (CI) were calculated to assess the relative risk of body constitutions and BMI.

## 3. Results

The baseline characteristics of the 3748 participants are listed (Table 1). The mean age of the total sample was 69.33 years. The proportion of subjects with habits of drinking and smoking was 16.78% and 18.94%, respectively. The prevalence of CAD, HTN, DM, and MetS was 11.18%, 42.58%, 16.84%, and 10.80%, respectively. The prevalence of overweight, obesity, and underweight in the total sample was 25.43%, 10.54%, and 2.48%, respectively. Significant differences were reported in weight, age, and drinking habits and the prevalence of HTN, DM, and MetS among the four groups categorized by BMI.

### 3.1. Univariate Analysis for BMI, Overweight, Obesity, and Underweight

**Figure 1:**
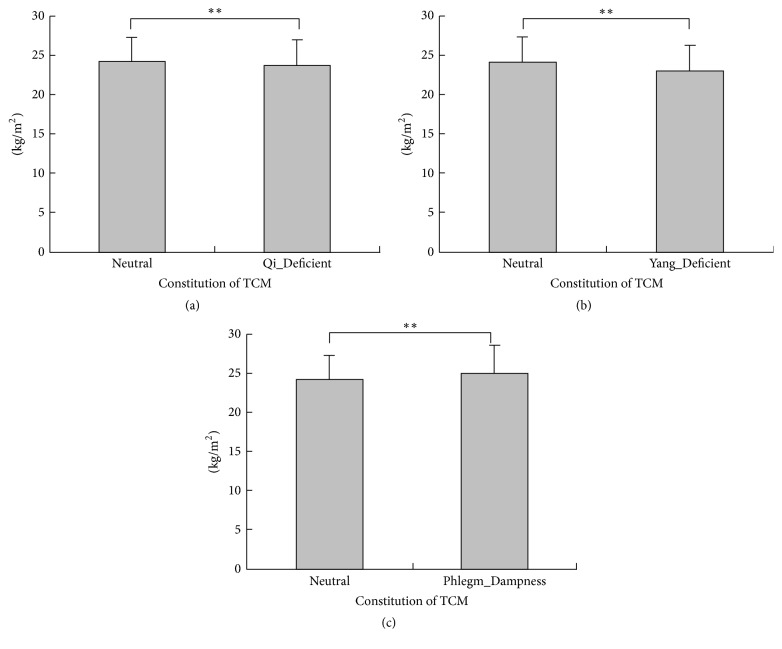
Comparison of body mass index among Neutral group and the other traditional Chinese medicine constitution groups. (a) The mean of body mass index was 24.24 kg/m^2^ and 23.66 kg/m^2^ in Neutral group and Qi_Deficient group (*P* < 0.001), respectively; (b) the mean of body mass index was 24.24 kg/m^2^ and 23.07 kg/m^2^ in Neutral group and Yang_Deficient group (*P* < 0.001), respectively; (c) the mean of body mass index was 24.24 kg/m^2^ and 25.10 kg/m^2^ in Neutral group and Phlegm_Dampness group (*P* < 0.001), respectively.  ^*∗∗*^Very significant.

**Figure 2:**
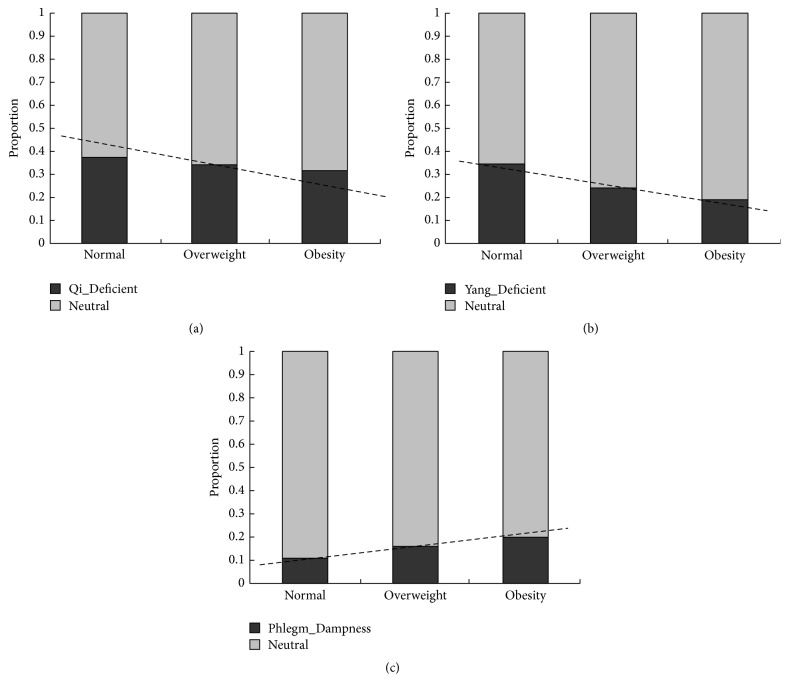
Distribution of normal BMI, overweight, and obesity among Neutral group and the other TCM constitution groups. (a) The proportion of normal BMI, overweight, and obesity was 37.52%, 34.07%, and 31.38% in Qi_Deficient group (*P* = 0.044 and *P* for trend = 0.043), respectively; (b) the proportion of normal BMI, overweight, and obesity was 34.49%, 23.80%, and 19.20% in Yang_Deficient group (*P* < 0.001 and *P* for trend <0.001), respectively; and (c) the proportion of normal BMI, overweight, and obesity was 10.62%, 15.54%, and 19.78% in Phlegm_Dampness group (*P* < 0.001 and *P* for trend <0.001), respectively.

**Figure 3:**
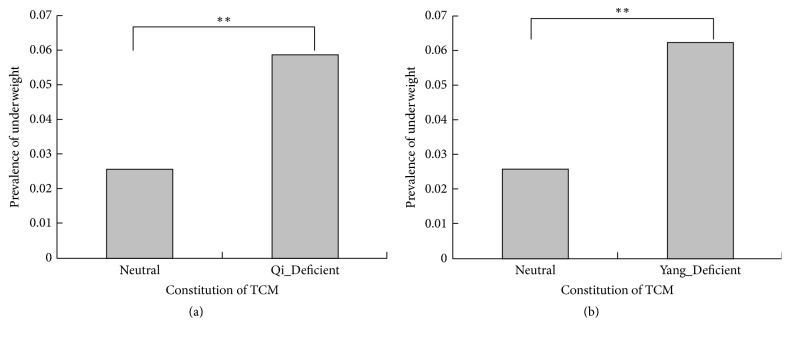
Comparison of prevalence of underweight among Neutral group and the other traditional Chinese medicine constitution groups. (a) The prevalence of underweight was 2.59% and 5.86% in Neutral group and Qi_Deficient group (*P* < 0.001), respectively; (b) the prevalence of underweight was 2.59% and 6.21% in Neutral group and Yang_Deficient group (*P* < 0.001), respectively.  ^*∗∗*^Very significant.

Univariate analyses showed that the Qi_Deficient, Yang_Deficient, and Phlegm_Dampness variables were associated with BMI types significantly (*P* value < 0.05 for three, Table 2). The mean of BMI in the Neutral, Qi_Deficient, Yang_Deficient, and Phlegm_Dampness groups was 24.24 kg/m^2^, 23.66 kg/m^2^, 23.07 kg/m^2^, and 25.10 kg/m^2^ (*P* < 0.001 for all, Figures 1(a)–1(c)), respectively. The proportions of normal BMI, overweight, and obesity were 37.52%, 34.07%, and 31.38% in the Qi_Deficient group (*P* = 0.044 and *P* for trend = 0.043, Figure 2(a)), respectively. The proportions were 34.49%, 23.80%, and 19.20% in the Yang_Deficient group (*P* < 0.001 and *P* for trend <0.001, Figure 2(b)), respectively, and 10.62%, 15.54%, and 19.78% in the Phlegm_Dampness group (*P* < 0.001 and *P* for trend <0.001, Figure 2(c)), respectively. Underweight participants accounted for 2.59%, 5.86%, and 6.21% in the Neutral, Qi_Deficient, and Yang_Deficient groups (*P* < 0.001 for all, Figures 3(a) and 3(b)), respectively. The univariate analysis revealed a negative correlation between the Qi_Deficient and Yang_Deficient groups and BMI, overweight, obesity, and underweight, whereas it indicated a positive correlation between Phlegm_Dampness and these outcomes.

### 3.2. Multiple Variable Analysis for BMI, Overweight, Obesity, and Underweight

The MLR detected significant associations of the Qi_Deficient, Yang_Deficient, and Phlegm_Dampness constitutions with the BMI outcomes, after controlling for potential confounding factors (*P* < 0.001 for three, Table 3). Moreover, the MLR with a shared reference of the normal weight BMI group reported that outcomes of overweight, obesity, and underweight were significant and independently associated with the Qi_Deficient and Yang_Deficient groups (*P* < 0.10 for all, Table 4), respectively. the results indicated that Phlegm_Dampness was associated with the outcomes of overweight and obesity (*P* < 0.01 for two) but was not associated with underweight (*P* = 0.948). No significant association was found in the other constitution groups (data not shown).

## 4. Discussion

The associations of the TCM constitutions with the overweight, obesity, and underweight BMI outcomes were analyzed for a sample of individuals from the Chinese population. Several studies on the body constitution in TCM have been conducted in recent years, and most of the study participants were from the central or western region of China [18–20]. Obesity has become a major global health challenge due to its increasing prevalence and associated health risks [21, 22]. Our data showed that 10.13% of adults suffered from obesity, similar to the figure reported for our sample. Much of the research has demonstrated that unhealthy lifestyles in terms of habits such as alcohol consumption lead to overweight and obesity, which in turn are closely related to many chronic diseases [23–25]. We used a standard questionnaire to evaluate the body constitution as per TCM in a large sample of Chinese from rural and urban communities of Shanghai. Moreover, to the best of our knowledge, this is the first study to employ the MLR approach of including a shared reference of normal weight to investigate the associations among the TCM body constitution types and the outcomes of overweight, obesity, and underweight. It is critical for integrative medicine practitioners to understand the clinical significance of TCM for these outcomes. This is partly because prevention (known in TCM as “Zhi Wei Bing”) is the one of the most important essences of TCM, indicating the preventive treatment of diseases. The body constitution in TCM could indicate the patients' overall condition and could form the basis of prevention (i.e., Zhi Wei Bing) theory; thus, it has attracted much attention from researchers and clinicians.

Our research yielded interesting findings. Overweight, obesity, and underweight outcomes were significant and independent and were negatively correlated with the Qi_deficient and Yang_Deficient groups. The univariate analysis indicated that the BMI outcomes were lower in the Qi_Deficient and Yang_Deficient groups as compared to the Neutral group (*P* < 0.05 for all). Moreover, multivariate analysis, controlling for potential confounders, reported that these two constitution types were significantly and independently associated with BMI and its outcomes (*P* < 0.010 for all). The most distinguished feature of the Yang_Deficiency type of constitution is that people of this type usually feel cold. People who are overweight and suffering from obesity have excessive subcutaneous fat, the function of which is to maintain body temperature and help them resist colder temperatures. Yanbo et al. made a similar conclusion, finding that a slim body was significantly positively correlated to the Yang_Deficiency type of constitution [26]. The study indicated that 2 genes were upregulated and 38 genes were downregulated significantly among individuals with the Qi_Deficiency type of constitution as compared with those having a Neutral constitution; further, the downregulated genes including the ATP binding gene, GTP binding gene, MHC protein binding gene, and AMP kinase were involved in ion transport, cholesterol synthesis, and fatty acid synthesis [27]. The downregulation of these genes might imply reduced heat production, a lack of appetite, and weight loss. However, there are also some contradictory research results showing a positive relationship between Qi_Deficiency and obesity [18, 28]. In view of the complexity of the body constitution in TCM, we consider the proportion of unbalanced constitution types in one person at the same time; accordingly, the Qi_Deficiency constitution could coexist with other constitution types in one person, thus leading to different results [29].

Another interesting finding was that the Phlegm_Dampness type of constitution was positively, significantly, and independently correlated with overweight or obesity, but it was not associated with underweight. Overweight people are phlegmatic according to the classical theories of TCM. Our results showed that the Phlegm_Dampness constitution is positively associated with obesity and overweight, which is consistent with the findings of previous research [30, 31]. The Phlegm_Dampness constitution type is very common in the study population. Several studies researched the differences between the Phlegm_Dampness and non-Phlegm_Dampness types from genome and single nucleotide polymorphisms (SNP), indicating that several genes and SNPs are significantly different between the Phlegm_Dampness and Neutral constitutions [32–34]. The identified genes are involved in enzyme activities and sterol transporter activities as well as in the process of lipid metabolism, cholesterol metabolism, brown fat cell differentiation, gluconeogenesis, and thermoregulation, indicating that an individual with Phlegm_Dampness constitution is susceptible to metabolic disorders including obesity [35]. Subjects with the Phlegm_Dampness constitution type usually manifest a series of comprehensive characteristics such as obesity, a preference for greasy food, large bellies, fatigue, and a slippery pulse [32]. Their constitution is affected by an initial endowment as well as environmental and lifestyle factors. Qi et al. found that greasy food, lack of exercise, irregular sleep, and smoking were all important determinants of the Phlegm_Dampness constitution [30]. Research in genomics has shown four upregulated genes and six downregulated genes in the Phlegm_Dampness constitution [35]. Qi et al. found the discrepancy between the Phlegm_Dampness constitution and non-Phlegm_Dampness constitution of obesity in ATP binding gene including Janus kinase 2, nuclear receptor protein, and CDC1 [36], providing molecular biological evidence for the association between the Phlegm_Dampness constitution and overweight and obesity. In clinical practice, we could apply the constitution of the Chinese medicine scale to estimate the risks of the Phlegm_Dampness constitution in terms of obesity and overweight and its related diseases. The constitutional theory of TCM recognizes individual differences from a synthetic and dynamic perspective based on morphological, functional, and psychological characteristics. Further research on the Phlegm_Dampness constitution is critical to enable the prediction and prevention of overweight, obesity, or related diseases.

This study has some limitations. On the one hand, the research sample was not recruited via a rigorous random sampling method, which means that the results are not sufficiently representative of the population in Shanghai. On the other hand, the factors affecting the constitution were not comprehensive, such as education, and psychological factors should be taken into account in future research.

## 5. Conclusion

Our findings provided evidence that the Qi_Deficient and Yang_Deficient constitution types were significantly and independently associated with the outcomes of overweight, obesity, and underweight. Phlegm_Dampness was positively, significantly, and independently correlated with the outcomes of overweight and obesity. The Qi_Deficient or Yang_Deficient participants were less frequently found to be overweight or obese as compared with participants with Neutral constitution. A higher prevalence of overweight or obesity was found in Phlegm_Dampness participants. These findings may provide insights into clinical practice toward the prevention and diagnosis of the overweight, obesity, and underweight health outcomes.

## Supplementary Material

Questionnaire of constitution of Chinese traditional medicine.

## Figures and Tables

**Table 1:** Baseline characteristics of participants.

Variable	Normal BMI	Overweight	Obesity	Underweight	Total	*P* value
Demographical information						
*N* (%)	2307 (61.55%)	953 (25.43%)	395 (10.54%)	93 (2.48%)	3748	—
Age (years)	69.37 ± 7.57	69.26 ± 7.34	68.71 ± 7.27	71.18 ± 9.12	69.33 ± 7.53	0.043
Gender (female)	1260 (54.61%)	499 (52.36%)	224 (56.71%)	56 (60.21%)	2039 (54.40%)	0.156
Height (cm)	161.28 ± 8.06	161.24 ± 8.08	160.34 ± 8.86	159.65 ± 7.93	161.26 ± 15.19	0.057
Weight (kg)	58.17 ± 7.68	68.48 ± 7.11	77.4 ± 9.01	43.91 ± 4.73	62.37 ± 10.82	<0.001
SBP (mmHg)	134.57 ± 22.62	135.8 ± 16.18	131.86 ± 16.55	134.67 ± 35.91	134.44 ± 19.98	0.948
DBP (mmHg)	64.5 ± 23.26	66.39 ± 22.9	78.64 ± 30.46	65 ± 13.45	67.12 ± 24.07	0.282
HR (bpm)	74.09 ± 14.21	74.26 ± 12.83	73.74 ± 12.23	76.45 ± 16.89	74.19 ± 13.73	0.405
Lifestyle						
Smoking (yes)	424 (18.38%)	181 (18.99%)	90 (22.78%)	15 (16.13%)	710 (18.94%)	0.148
Alcohol (yes)	368 (15.95%)	173 (18.15%)	82 (20.76%)	6 (6.45%)	629 (16.78%)	0.004
Dietary (unbalance)	52 (2.25%)	21 (2.20%)	13 (3.29%)	4 (4.30%)	90 (2.40%)	0.127
Exercise (yes)	1441 (62.46%)	577 (60.55%)	233 (58.99%)	50 (53.76%)	2301 (61.39%)	0.18
Medical history						
HTN (yes)	866 (37.54%)	485 (50.89%)	225 (56.96%)	20 (21.51%)	1596 (42.58%)	<0.001
DM (yes)	333 (14.43%)	192 (20.15%)	99 (25.06%)	7 (7.53%)	631 (16.84%)	<0.001
CAD (yes)	261 (11.31%)	104 (10.91%)	39 (9.87%)	15 (16.13%)	419 (11.18%)	0.343
MetS (yes)	10 (0.43%)	277 (29.06%)	118 (29.87%)	0 (0%)	405 (10.80%)	<0.001

*Note.* SBP: systolic blood pressure; DBP: diastolic blood pressure; HR: heart rate; HTN: hypertension; DM: diabetes mellitus; CAD: coronary artery disease; and MetS: metabolic syndrome.

**Table 2:** Comparison of distribution of different body mass index among Neutral group and the other TCM constitution groups.

Variable	Normal BMI	Overweight	Obesity	Underweight	Total	*P* value
Neutral	1204	538	224	34	2010	—
Qi_Deficient	723	278	103	49	1153	<0.001
Yang_Deficient	634	169	54	45	902	<0.001
Yin_Deficient	210	81	32	7	330	0.621
Phlegm_Dampness	143	99	55	5	302	<0.001
Damp_Heat	76	30	17	1	124	0.385
Blood_Stasis	44	20	9	1	74	0.992
Qi_Stagnation	31	13	8	1	53	0.832
Special	49	25	8	3	85	0.511

*Note.* TCM: traditional Chinese medicine.

**Table 3:** Multinomial logistic analysis to include TCM constitution for BMI.

Variable	Beta	S.E	*t* value	*P* value	95% CI for Beta
Qi_Deficient	−0.578	−0.087	−4.85	<0.001	−0.812–−0.345
Yang_Deficient	−1.112	−0.162	−8.794	<0.001	−1.36–−0.864
Phlegm_Dampness	0.85	0.089	4.348	<0.001	0.466–1.233

*Note.* Multiple variable analysis adjusted for age, gender, exercise, dietary, smoking, alcohol, HTN, DM, and CAD; TCM: traditional Chinese medicine and BMI: body mass index.

**Table 4:** Multinomial logistic regression analysis to include TCM constitution for overweight, obesity, and underweight.

TCM constitution	Outcome	Beta	S.E	*P* value	OR	95% CI
Qi_Deficient	Overweight	−0.149	0.085	0.098	0.862	0.785–1.018
	Obesity	−0.312	0.133	0.019	0.732	0.564–0.951
	Underweight	0.784	0.243	0.001	2.191	1.361–3.529

Yang_Deficient	Overweight	−0.509	0.104	<0.001	0.601	0.49–0.737
	Obesity	−0.781	0.165	<0.001	0.458	0.332–0.633
	Underweight	0.812	0.246	0.001	2.253	1.391–3.65

Phlegm_Dampness	Overweight	0.448	0.142	0.002	1.565	1.184–2.068
	Obesity	0.745	0.179	<0.001	2.107	1.485–2.991
	Underweight	−0.035	0.542	0.948	0.965	0.334–2.793

*Note.* Multiple variable analysis adjusted for age, gender, exercise, dietary, smoking, alcohol, HTN, DM, and CAD; TCM: traditional Chinese medicine.

## Abbreviations

TCMC: Traditional Chinese medicine constitution
SBP: Systolic blood pressure
DM: Diabetes mellitus
CAD: Coronary artery disease
MetS: Metabolic syndrome
HTN: Hypertension
BMI: Body mass index
DM: Diabetes mellitus
OGTT: Oral glucose tolerance test
MR: Multivariable regression
OR: Odds ratios
CI: Confidence intervals.

## Data Access

The datasets generated and/or analysed during the current study are not publicly available due to private information but are available from the corresponding author on reasonable request. Datasets are from the study whose authors may be contacted at Center of Bioinformatics and Biostatistics, Institutes of Integrative Medicine, Fudan University.
