# Supplementary material for: A Study of Traditional Chinese Medicine Body Constitution Associated with Overweight, Obesity, and Underweight
Source: Evid Based Complement Alternat Med. 2017 Oct 19;2017:7361896. doi: 10.1155/2017/7361896 (PMC5671717; doi:10.1155/2017/7361896)
Supplement: Supplementary file 1 — Questionnaire of constitution of Chinese traditional medicine. [file 7361896.f1.doc]

**Constitution in Chinese Medicine Questionnaire**

In mainland China, the Constitution in Chinese Medicine Questionnaire (CCMQ) was established by Wang et al. Sixty items were applied to measure the nine constitutions (Table.1-9): Neutral (a normal constitution), Qi-deficiency constitution, Yang-deficiency constitution, Yin-deficiency constitution, Phlegm-dampness constitution, Damp-heat constitution, Stagnant-blood constitution, Stagnant-Qi constitution, and Inherited-special constitution.

**Assessing Methods**

Firstly, answer the 60 questions. Secondly, every question is attached to 5 scores and then calculated the total scores and do some adjustments. Finally, estimate which constitution is involved according to the above scores.

Total scores (TS) are scores of every item added up. The adjusted scores (AS) are｛[ (TS - the number of items)/( the number of items×4)]×100｝.

**Assessing Criterion**

Neutral constitution is a normal constitution, while the 8 other unbalanced constitution is too excessive or too less. The system of evaluation criteria is as followed.

| Types of constitution | Criteria | Results |
| --- | --- | --- |
| Neutral constitution | AS≧60 | Yes |
|  | AS of the 8 other unbalanced constitution﹤30 |  |
|  | AS≧60 | Prone to “Yes” |
|  | AS of the 8 other unbalanced constitution﹤40 |  |
|  | Not meet the above conditions | NO |
| Unbalanced constitutions | AS≧40 | Yes |
|  | AS 30~39 | Prone to “Yes” |
|  | AS﹤30 | NO |

**Examples**

*Example 1* After finishing the questionnaire, one person got the following scores: Qi-deficiency constitution 56 scores, Yang-deficiency constitution 27 scores, Yin-deficiency constitution 25 scores, Phlegm-dampness constitution 12 scores, Damp-heat constitution 15 scores, Stagnant-blood constitution 20 scores, Stagnant-qi constitution 18 scores, Inherited-special constitution 10 scores and Neutral constitution 75 scores. According to the criteria, although the AS of Neutral constitution are more than 60 scores, the AS of the 8 other unbalanced constitution are not all less than 40 scores, of which the AS of Qi-deficiency constitution is more than 40 scores. Then, we finally drew the conclusion that the person had Qi-deficiency constitution.

*Example 2* After finishing the questionnaire, one person got the scores: Qi-deficiency constitution 16 scores, Yang-deficiency constitution 27 scores, Yin-deficiency constitution 25 scores, Phlegm-dampness constitution 32 scores, Damp-heat constitution 18 scores, Stagnant-blood constitution 10 scores, Stagnant-qi constitution 18 scores, Inherited-special constitution 10 scores and Neutral constitution 75 scores. On the basis of the criteria, the AS of Neutral constitution are more than 60, but the the AS of Phlegm-dampness constitution are between 30 and 39 scores. Accordingly, we finally pointed out that the person had Neutral constitution with the leaning to Qi-deficiency constitution.

**Tables**

**Table1. Yang-deficiency Constitution**

| According to the nearly a year of experience and feeling, please answer the following questions | NO  (not at all) | Scarcely  (little) | Sometimes  (some) | Often  ([comparatively](../../../../D:/Youdao/Dict/6.3.69.8341/resultui/frame/javascript:void(0)%3B)) | Always  (very) |
| --- | --- | --- | --- | --- | --- |
| (1) Did your hands or feet feel cold or clammy? | 1 | 2 | 3 | 4 | 5 |
| (2) Did you feel cold easily in your abdomen, back, lower back or knees? | 1 | 2 | 3 | 4 | 5 |
| (3) Were you sensitive to cold and tend to wear more clothes than others? | 1 | 2 | 3 | 4 | 5 |
| (4) Did you feel more vulnerable to the cold than others(winter coldness, air conditioners, fans, etc.)? | 1 | 2 | 3 | 4 | 5 |
| (5) Did you catch colds more easily than others? | 1 | 2 | 3 | 4 | 5 |
| (6) Did you feel uncomfortable when you drank or ate something cold or do you avoid to drinking or eating something cold? | 1 | 2 | 3 | 4 | 5 |
| (7) Did you easily contract diarrhea when you were exposed to cold or eat(or drink)something cold? | 1 | 2 | 3 | 4 | 5 |
| Results：□Yes   □ Prone to “Yes”   □NO | | | | | |

Table2. Yin-deficiency Constitution

| According to the nearly a year of experience and feeling, please answer the following questions | NO  (not at all) | Scarcely  (little) | Sometimes  (some) | Often  ([comparatively](../../../../D:/Youdao/Dict/6.3.69.8341/resultui/frame/javascript:void(0)%3B)) | Always  (very) |
| --- | --- | --- | --- | --- | --- |
| (1)Do the palms of your hands or soles ofyour feet feel hot? | 1 | 2 | 3 | 4 | 5 |
| (2)Did your body and face feel hot? | 1 | 2 | 3 | 4 | 5 |
| (3) Did your skin or lips feel dry? | 1 | 2 | 3 | 4 | 5 |
| (4)Were your lips redder than others? | 1 | 2 | 3 | 4 | 5 |
| (5) Did you get constipated easily or have dry stools? | 1 | 2 | 3 | 4 | 5 |
| (6) Did you get hot flashes? | 1 | 2 | 3 | 4 | 5 |
| (7) Did your eyes feel dry and use eye drops? | 1 | 2 | 3 | 4 | 5 |
| (8)Did you sweat easily when you had a slightly increased physical activity? | 1 | 2 | 3 | 4 | 5 |
| Results：□Yes   □ Prone to “Yes”   □NO | | | | | |

**Table3. Qi-deficiency Constit**ution

| According to the nearly a year of experience and feeling, please answer the following questions | NO  (not at all) | Scarcely  (little) | Sometimes  (some) | Often  ([comparatively](../../../../D:/Youdao/Dict/6.3.69.8341/resultui/frame/javascript:void(0)%3B)) | Always  (very) |
| --- | --- | --- | --- | --- | --- |
| (1) Did you get tired easily? | 1 | 2 | 3 | 4 | 5 |
| (2)Did you suffer from shortness of breath? | 1 | 2 | 3 | 4 | 5 |
| (3) Did you get palpitations? | 1 | 2 | 3 | 4 | 5 |
| (4) Did you get dizziness easily or become giddy when standing up? | 1 | 2 | 3 | 4 | 5 |
| (5) Did you catch colds more easily than others? | 1 | 2 | 3 | 4 | 5 |
| (6) Didyou prefer quietness and do not like to talk? | 1 | 2 | 3 | 4 | 5 |
| (7) Do you feel weak when talking? | 1 | 2 | 3 | 4 | 5 |
| (8) Did you sweat easily when you had a slightly increased physical activity? |  |  |  |  |  |
| Results：□Yes   □ Prone to “Yes”   □NO | | | | | |

Table4. Damp-heat Constitution

| According to the nearly a year of experience and feeling, please answer the following questions | NO  (not at all) | Scarcely  (little) | Sometimes  (some) | Often  ([comparatively](../../../../D:/Youdao/Dict/6.3.69.8341/resultui/frame/javascript:void(0)%3B)) | Always  (very) |
| --- | --- | --- | --- | --- | --- |
| (1) Did you feel chest or stomach stuffiness? | 1 | 2 | 3 | 4 | 5 |
| (2) Did your body feel heavy or lethargic? | 1 | 2 | 3 | 4 | 5 |
| (3) Was your stomach/belly flabby? | 1 | 2 | 3 | 4 | 5 |
| (4) Did you have an excessively oily forehead and/or T—zone? | 1 | 2 | 3 | 4 | 5 |
| (5) Did you have upper eyelid swelling? | 1 | 2 | 3 | 4 | 5 |
| (6) Did your mouth feel sticky? | 1 | 2 | 3 | 4 | 5 |
| (7) Did you have lots of phlegm，especially in your throat? | 1 | 2 | 3 | 4 | 5 |
| (8) Did your tongue have a thick coating? | 1 | 2 | 3 | 4 | 5 |
| Results：□Yes   □ Prone to “Yes”   □NO | | | | | |

Table5. Damp-heat Constitution

| According to the nearly a year of experience and feeling, please answer the following questions | NO  (not at all) | Scarcely  (little) | Sometimes  (some) | Often  ([comparatively](../../../../D:/Youdao/Dict/6.3.69.8341/resultui/frame/javascript:void(0)%3B)) | Always  (very) |
| --- | --- | --- | --- | --- | --- |
| (1) Did your nose or your face feel greasy, oily, or shiny? | 1 | 2 | 3 | 4 | 5 |
| (2) Did you get acne or sores easily? | 1 | 2 | 3 | 4 | 5 |
| (3) Did you have bitterness or a strange taste in your mouth? | 1 | 2 | 3 | 4 | 5 |
| (4) Did you pass sticky stools and /or feelthatyour bowel movement is incomplete? | 1 | 2 | 3 | 4 | 5 |
| (5) Did your urethral canal feel hot when you urinated, or did your urine have a dark color? | 1 | 2 | 3 | 4 | 5 |
| (6) Was your vaginal discharge yellowish ly for female interviewees)? | 1 | 2 | 3 | 4 | 5 |
| (7) Was your scrotum always wet(only for male interviewees)? | 1 | 2 | 3 | 4 | 5 |
| Results：□Yes   □ Prone to “Yes”   □NO | | | | | |

**Table6. Stag**nant-blood Constitution

| According to the nearly a year of experience and feeling, please answer the following questions | NO  (not at all) | Scarcely  (little) | Sometimes  (some) | Often  ([comparatively](../../../../D:/Youdao/Dict/6.3.69.8341/resultui/frame/javascript:void(0)%3B)) | Always  (very) |
| --- | --- | --- | --- | --- | --- |
| (1) Did black or purple bruises appear on your skin for no reason? | 1 | 2 | 3 | 4 | 5 |
| (2) Did you have visible capillary/threadveinson yourcheeks? | 1 | 2 | 3 | 4 | 5 |
| (3) Did you feel pain somewhere in your body? | 1 | 2 | 3 | 4 | 5 |
| (4) Did you have a dark face or get brown spots easily? | 1 | 2 | 3 | 4 | 5 |
| (5) Did you get dark circles under the eyes easily? | 1 | 2 | 3 | 4 | 5 |
| (6) Did you forget things easily? | 1 | 2 | 3 | 4 | 5 |
| (7) Did your lips darker, more blue or purpie than usual? | 1 | 2 | 3 | 4 | 5 |
| Results：□Yes   □ Prone to “Yes”   □NO | | | | | |

Table7. Inherited-special Constitution

| According to the nearly a year of experience and feeling, please answer the following questions | NO  (not at all) | Scarcely  (little) | Sometimes  (some) | Often  ([comparatively](../../../../D:/Youdao/Dict/6.3.69.8341/resultui/frame/javascript:void(0)%3B)) | Always  (very) |
| --- | --- | --- | --- | --- | --- |
| (1) Did you sneeze even when you did not have a cold? | 1 | 2 | 3 | 4 | 5 |
| (2) Did you have runny or stuffy nose even when you did not have a cold? | 1 | 2 | 3 | 4 | 5 |
| (3) Did you cough due to seasonal change, temperature change, or unpleasant odor? | 1 | 2 | 3 | 4 | 5 |
| 1. Did you have allergies?(E.g. Medicine, food, odors, pollen, pet dander, orduring seasonal or weather change etc.)? | 1 | 2 | 3 | 4 | 5 |
| (5) Did your skin get hives/urticaria easily? | 1 | 2 | 3 | 4 | 5 |
| (6) Did your skin have purpura(purple spots, ecchymosis)due to allergies? | 1 | 2 | 3 | 4 | 5 |
| (7) Did you skin turn red and show traces when you scratched it? | 1 | 2 | 3 | 4 | 5 |
| Results：□Yes   □ Prone to “Yes”   □NO | | | | | |

Table8. Stagnant-qi Constitution

| According to the nearly a year of experience and feeling, please answer the following questions | NO  (not at all) | Scarcely  (little) | Sometimes  (some) | Often  ([comparatively](../../../../D:/Youdao/Dict/6.3.69.8341/resultui/frame/javascript:void(0)%3B)) | Always  (very) |
| --- | --- | --- | --- | --- | --- |
| (1) Did you feel gloomy and depressed? | 1 | 2 | 3 | 4 | 5 |
| (2) Do you get anxious and worried easily? | 1 | 2 | 3 | 4 | 5 |
| (3) Did you feel sensitive，vulnerable or emotionally upset? | 1 | 2 | 3 | 4 | 5 |
| (4) Were you easily scared or frightened? | 1 | 2 | 3 | 4 | 5 |
| (5) Did you feel chest or stomach stuffiness? | 1 | 2 | 3 | 4 | 5 |
| (6) Did you sigh for no reason? | 1 | 2 | 3 | 4 | 5 |
| (7) Did your throat feel strange(i.e, likesomething was stuck or there was a lump in your throat)? | 1 | 2 | 3 | 4 | 5 |
| Results：□Yes   □ Prone to “Yes”   □NO | | | | | |

Table9. Neutral (a normal) constitution

| According to the nearly a year of experience and feeling, please answer the following questions | NO  (not at all) | Scarcely  (little) | Sometimes  (some) | Often  ([comparatively](../../../../D:/Youdao/Dict/6.3.69.8341/resultui/frame/javascript:void(0)%3B)) | Always  (very) |
| --- | --- | --- | --- | --- | --- |
| (1) Were you energetic? | 1 | 2 | 3 | 4 | 5 |
| (2) Did you get tired easily? | 1 | 2 | 3 | 4 | 5 |
| (3) Do you feel weak when talking? | 1 | 2 | 3 | 4 | 5 |
| (4) Did you feel gloomy and depressed? | 1 | 2 | 3 | 4 | 5 |
| (5) Did you feel more vulnerable to the cold than others (winter coldness, air conditioners, fans, etc.)? | 1 | 2 | 3 | 4 | 5 |
| (6) Could you adapt yourself to external natural or social environment change? | 1 | 2 | 3 | 4 | 5 |
| (7) Did you suffer from insomnia? | 1 | 2 | 3 | 4 | 5 |
| (8) Did you forget things easily? |  |  |  |  |  |
| Results：□Yes   □ Prone to “Yes”   □NO | | | | | |

**Acknowledgement**

As translating the Chinese version of CCMQ, we referred to the English version of CCMQ made by *JING Hui-ru et al*[1]*.*

**Reference:**

JING Hui-ru，WANG Ji，WANG Qi. Preliminary Compiling of English Version of Constitution in Chinese Medicine Questionnaire. *J ANHUI UNIV CHINESE ME* 2015: 34(5): 21-25.

STROBE Statement—checklist of items that should be included in reports of observational studies

|  | Page | Recommendation |
| --- | --- | --- |
| **Title and abstract** | 1-5 | (*a*) Indicate the study’s design with a commonly used term in the title or the abstract |
|  |  | (*b*) Provide in the abstract an informative and balanced summary of what was done and what was found |
| Introduction | | |
| Background/rationale | 6-7 | Explain the scientific background and rationale for the investigation being reported |
| Objectives | 7 | State specific objectives, including any prespecified hypotheses |
| Methods | | |
| Study design | 7-8 | Present key elements of study design early in the paper |
| Setting | 7-8 | Describe the setting, locations, and relevant dates, including periods of recruitment, exposure, follow-up, and data collection |
| Participants | 7-8 | (*a*) *Cross-sectional study*—Give the eligibility criteria, and the sources and methods of selection of participants |
|  |  |  |
| Variables | 7-8 | Clearly define all outcomes, exposures, predictors, potential confounders, and effect modifiers. Give diagnostic criteria, if applicable |
| Data sources/ measurement | 7-8 | For each variable of interest, give sources of data and details of methods of assessment (measurement). Describe comparability of assessment methods if there is more than one group |
| Bias | 8 | Describe any efforts to address potential sources of bias |
| Study size | 8 | Explain how the study size was arrived at |
| Quantitative variables | 8 | Explain how quantitative variables were handled in the analyses. If applicable, describe which groupings were chosen and why |
| Statistical methods | 8-9 | (*a*) Describe all statistical methods, including those used to control for confounding |
|  |  | (*b*) Describe any methods used to examine subgroups and interactions |
|  |  | (*c*) Explain how missing data were addressed |
|  |  | (*d*) *Cross-sectional study*—If applicable, describe analytical methods taking account of sampling strategy |
|  |  | (*e*) Describe any sensitivity analyses |

Continued on next page

| Results | | |
| --- | --- | --- |
| Participants | 7-8 | (a) Report numbers of individuals at each stage of study—eg numbers potentially eligible, examined for eligibility, confirmed eligible, included in the study, completing follow-up, and analysed |
|  |  | (b) Give reasons for non-participation at each stage |
|  |  | (c) Consider use of a flow diagram |
| Descriptive data | 9 | (a) Give characteristics of study participants (eg demographic, clinical, social) and information on exposures and potential confounders |
|  |  | (b) Indicate number of participants with missing data for each variable of interest |
|  |  | (c) *Cohort study*—Summarise follow-up time (eg, average and total amount) |
| Outcome data | 9 | *Cohort study*—Report numbers of outcome events or summary measures over time |
|  |  | *Case-control study—*Report numbers in each exposure category, or summary measures of exposure |
|  |  | *Cross-sectional study—*Report numbers of outcome events or summary measures |
| Main results | 9-11 | (*a*) Give unadjusted estimates and, if applicable, confounder-adjusted estimates and their precision (eg, 95% confidence interval). Make clear which confounders were adjusted for and why they were included |
|  |  | (*b*) Report category boundaries when continuous variables were categorized |
|  |  | (*c*) If relevant, consider translating estimates of relative risk into absolute risk for a meaningful time period |
| Other analyses | 11 | Report other analyses done—eg analyses of subgroups and interactions, and sensitivity analyses |
| Discussion | | |
| Key results | 11 | Summarise key results with reference to study objectives |
| Limitations | 14-15 | Discuss limitations of the study, taking into account sources of potential bias or imprecision. Discuss both direction and magnitude of any potential bias |
| Interpretation | 11-14 | Give a cautious overall interpretation of results considering objectives, limitations, multiplicity of analyses, results from similar studies, and other relevant evidence |
| Generalisability | 12-14 | Discuss the generalisability (external validity) of the study results |
| Other information | | |
| Funding | 18 | Give the source of funding and the role of the funders for the present study and, if applicable, for the original study on which the present article is based |

*Give information separately for cases and controls in case-control studies and, if applicable, for exposed and unexposed groups in cohort and cross-sectional studies.

**Note:** An Explanation and Elaboration article discusses each checklist item and gives methodological background and published examples of transparent reporting. The STROBE checklist is best used in conjunction with this article (freely available on the Web sites of PLoS Medicine at http://www.plosmedicine.org/, Annals of Internal Medicine at http://www.annals.org/, and Epidemiology at http://www.epidem.com/). Information on the STROBE Initiative is available at www.strobe-statement.org.
